# Cognitive flexibility in aging: the impact of age range and task difficulty on local switch costs in task switching

**DOI:** 10.3389/fnagi.2025.1619441

**Published:** 2025-08-26

**Authors:** Tara Radović, Sebastian Kübler, Rafael Mikolajczyk, Alexander Kluttig, Viktoria Maydych, Torsten Schubert

**Affiliations:** ^1^Institute for Psychology, Martin Luther University Halle-Wittenberg, Halle (Saale), Germany; ^2^Department of Psychology, Humboldt University of Berlin, Berlin, Germany; ^3^Institute for Medical Epidemiology, Biometrics, and Informatics, Interdisciplinary Centre for Health Sciences, Martin Luther University Halle-Wittenberg, Halle (Saale), Germany

**Keywords:** local switch cost, aging, cognitive flexibility, task difficulty, processing speed, frontal lobe hypothesis

## Abstract

**Introduction:**

Previous studies provided inconclusive results regarding the effects of aging on the ability to flexibly switch between task rules (local switch cost). The goal of the present study was to investigate the influence of age on the local switch costs at two levels of difficulty (easy task switching: two task rules vs. difficult task switching: four task rules).

**Methods:**

The local switch costs, i.e., reaction time and error differences between trials with a task switch and task repetition relative to the previous trial, were compared in a group of young adults (19 to 33 years) and three groups of older adults (64–72; 73–80; 82–97 years).

**Results:**

The analysis of the switch costs showed significantly higher switch costs of the three groups of older adults compared to the younger adults and the effect was more pronounced in the difficult task switching than in the easy task switching. At the same time, there were no clear differences in the local switch costs between the three groups of older adults.

**Discussion:**

The results showed that even after the age-related slowdown was taken into account, age differences in local switch costs will emerge when the age range of older adults is extended and task difficulty is sufficiently high. These findings contribute to our understanding of how and when age differences in cognitive flexibility emerge and suggest that complex multitasking environments may disproportionately challenge older adults.

## Introduction

Studies in the domain of cognitive aging often investigated the role of executive functions in age-related performance decline in various cognitive tasks (Kray et al., 2002). Namely, executive functions are higher-order control processes that coordinate and regulate behavior during performance of cognitive tasks (Miyake et al., 2000; Strobach et al., 2012, 2015). Importantly, the concept of executive control is not a unitary concept and includes different types of processes such as updating information, inhibition of irrelevant information, and switching between tasks (Miyake et al., 2000). It is established that executive control functions are negatively influenced by age (e.g., Hasher and Zacks, 1988; Mayr et al., 1996) and different explanations of these age-related changes were proposed.

For example, the processing-speed hypothesis (Salthouse, 1996, 2019) assumes that the speed of processing is a main constraint associated with older age. According to this view, slower processing takes place at perceptual level, cognitive level and motor-response level, leading to cognitive decline. Namely, slower processing can leave insufficient time for processing task components, especially under time constraints. This can cause incomplete early-stage processing and decay of relevant information that propagates to later processing stages thus impairing the integration of information across the whole processing chain. Consequently, as this incomplete processing in the early stages propagates and accumulates at later processing stages, slower processing of task components impairs performance in complex tasks more than in simple tasks. This, in turn, results in general cognitive impairments in older age, which are reflected in lower cognitive performance (e.g., in terms of absolute response times and more errors) of older adults, especially when the tasks are complex. Evidence for the processing-speed hypothesis as an explanation for age-related cognitive decline was provided by many previous studies (e.g., Salthouse, 1993; Salthouse et al., 1996). For example, in a study done by Salthouse (1993) various tests of perceptual speed, motor speed, memory and cognition were administered in adults ranging in age between 20 and 70 years old. When different age groups were equated at the level of basic processing speed, results of a mediation analysis revealed that between 80% and 100% of the age-related differences in memory and cognition tasks were eliminated.

Other theories propose that cognitive decline is accompanied by specific impairments in executive control processes such as inhibition (Hasher and Zacks, 1988) and task switching (Mayr, 2001; Toovey et al., 2021). In this case, age-related decline in performance is assumed to be more pronounced under certain task conditions (e.g., that involve interference processing and, thus, require executive control processes) compared to others (e.g., where no such interference is present and efficient task processing relies less on cognitive control), due to specific deficits in the cognitive control process (which are involved in dealing with that interference). Often such function-specific age-related impairments are explained by differential effects of aging on separate subsystems within the frontal cortex (e.g., Mitchell et al., 2023; Pergher et al., 2019; Phillips and Della Sala, 1998), which supports executive control functions and higher order cognitive processes (e.g., Braver et al., 2001; West, 1996). While there is evidence for age-specific effects in the domains of inhibition and working memory, the results of previous studies seem to be mixed when it comes to the effects of age on task-switching ability (Mayr and Liebscher, 2001; Mayr, 2001; Meiran et al., 2001; Kray et al., 2002). There are several possible reasons for this, including the fact that many previous studies on age-related changes in task-switching compared young adults with relatively young older adults, which may have been too narrow a range to detect consistent effects. Therefore, the current study aims to elucidate in more detail the effect of age on task-switching processes.

In the task-switching paradigm, participants are typically asked to execute a series of potentially alternating tasks, which require application of different rules, e.g., binary decisions or mental operations, in relation to different stimuli in close succession. Depending on a cue or a specific property of the presented stimulus, at each trial participants are required to apply a specific task rule for the task at hand. For example, in the classical task-switching study of Rogers and Monsell (1995) a character pair consisting of a letter and a number was presented moving clockwise within a frame of four-square boxes. Participants were told to perform the letter task (consonant/vowel task) when the character pair appeared in the upper part of the screen or the digit task (odd/even) when the character pair appeared at the bottom part of the screen. Critically, these different tasks are typically completed in single-task blocks including only one task rule (repetition trials only) and in mixed-task blocks in which occasional switching between different task rules is required (repetition and switching trials are mixed). Based on the performance in these two types of blocks, global and local switch costs are typically measured. While global switch costs are calculated as a difference in response times (RT) between repetition trials in the mixed-block and repetition trials in the single block local switch costs are calculated as a difference in RTs in switching trials and repetition trials within the mixed block. The switch costs can be explained by different theoretical accounts, such as task-set reconfiguration (e.g., Ulrich and Kliegl, 2000; Rogers and Monsell, 1995) and task-set inertia (e.g., Allport et al., 1994). Task set reconfiguration assumes top-down executive control processes that take place on the switching trials, which include active maintenance of task goal, task rules and stimulus-response mappings, as well as active inhibition of the previous task which is no longer relevant. On the other hand, task-inertia assumes a passive, bottom-up interference from the previously completed trial resulting from incomplete deactivation of that previous task and causing increased costs in the current (switching) trial.

Importantly, while global switch costs were shown to be reliably affected by aging, results of previous studies on local switch costs are rather inconsistent (Idowu and Szameitat, 2023; Kray et al., 2002; Kray and Lindenberger, 2000; Mayr, 2001; Meiran et al., 2001; Reimers and Maylor, 2005). For example, Kray and Lindenberger (2000), investigated switch costs in young adults (mean age 29.6 years), middle-aged adults (mean age 50.3 years), and older adults (mean age 69.5). In this experiment, participants had to classify geometric figures according to shape (triangle vs. rectangle) or color (gray vs. colored). The study did not find any differences in local switch costs between the groups. This finding suggests that the capability to flexibly reconfigure the cognitive system from one task to the next remains intact across age. Similarly, Reimers and Maylor (2005) investigated age-related changes in task switching across the life span (10–66 years old). In this study, participants were presented face stimuli and asked to perform either a gender classification task (male vs. female) or an emotion classification task (sad vs. happy). Also here, local switch costs remained relatively stable across adulthood (above age 18) and became only tendentially more pronounced in the age group of 61–66 years. In line with this possibility, other studies (e.g., Mayr, 2001) suggested that the differences in local switch costs start to emerge when the group of younger participants is compared to the group of older participants (above 70 years old). For example, Mayr (2001) investigated switching between shape and color classification tasks in older adults (mean age 71 years) and younger adults (mean age 20.1 years old). The results revealed large age differences in global switch costs, while local switch costs were also affected but only moderately. Thus, it is tempting to assume that an age-related changes of local switch costs might start to emerge in a clearer fashion rather at a later age, i.e., as the mean age of the older group is above 70 years old, which is to be investigated in the current study.

To address the inconsistencies in the results of the previous studies on the effects of aging on local switch costs, we conducted the present study. We focused on this aspect of task switching as it reflects cognitive flexibility, i.e., activation and de-activation of task representations, which is necessary for dealing with changing contexts in everyday life (Chen and Hsieh, 2023). As mentioned above, previous studies that failed to find significant effects of aging on local switch costs mainly focused on a rather restricted age range, for example by comparing performance between young adults (up to 30 years old) and young-older adults (average age up to 69 years old). Therefore, if age-related changes in task-switching ability take place over a longer period, the age range of participants in the previous studies was relatively narrow for the effect to emerge consistently. Thus, by extending the age range to even older adults (i.e., exceeding the age of 70 years and more) we aim to test whether the age effects on local switch costs would potentially start to emerge at a later age.

Moreover, it is plausible to assume that older participants were not sufficiently challenged by the difficulty of task switching in previous studies leaving sufficient cognitive resources available for efficient switching between the tasks. Thus, in the present study we also included different difficulty levels of task switching. In fact, leaving sufficient cognitive resources available for efficient deactivation and activation of task sets might have resulted in intact local switch costs also in older adults. Indeed, when the difficulty of task switching was increased by introducing more than two task rules, age differences between older adults and younger adults in local switch costs were more pronounced (Kray et al., 2002).

Hence, in the current study, we aim to account for the possibility that local switch costs might come to light rather at later ages (above 70 years) and especially under sufficient cognitive load, i.e., when participants are required to operate at an upper cognitive limit. For this purpose, in the present study, besides the control group of young adults, we especially included groups of older participants covering a larger age range, i.e., between 64 and 97 years and divided them into three groups: the young old group (64–72), the older-old group (73–80), and the oldest-old group (82–97). Admittedly, previous studies in the domain of cognitive aging used different classification criteria for grouping older participants, which might also have contributed to the observation of inconsistent findings on potential age effects on cognitive functions. Here, we employed a classification system roughly corresponding to the propositions of the World Health Organization (World Health Organization [WHO], 2015). Moreover, to manipulate the amount of cognitive demands in task switching, we compared participants’ performance in an easy task-switching version (switching between two task rules) and a difficult task-switching version (switching between four task rules).

If the age range of older participants and difficulty of task switching are decisive factors to disclose potential age-related differences, we should find larger local switch costs across the older age range. More specifically, we expect that the difference between switching and repetition trials (i.e., local switch cost) in response times and error rates will be larger in the three groups of older participants compared to the group of young participants. Moreover, we expect that the difference in local switch costs to be greater in the group of young-old participants than in the group of older-old participants, as well as in the group of older-old than in the oldest-old group. This hypothesis stands in line with the assumption that aging has specific detrimental effects on executive control processes involved in task switching (e.g., Braver et al., 2001; Phillips and Della Sala, 1998; West, 1996).

Alternatively, if task switching ability is not specifically and additionally impaired by aging, we expect the following pattern of results. Namely, we expect to find longer response times and more errors in the switching trials compared to the repetition trials in line with the previous studies (e.g., Monsell, 2003). Critically, aging factor will be related to longer response times and more errors in both switching trials and repetition trials to the same extent meaning that the difference between switching and repetition trials (i.e., local switch cost) should not further differ between different age groups (Chen and Hsieh, 2023; Wasylyshyn et al., 2011). Nonetheless, we expect that the differences in local switch costs will be more pronounced in the difficult condition compared to the easy condition. This expectation would be in line with the predictions of the processing speed hypothesis (Salthouse, 1996), which assumes that aging leads to a general slowdown in processing which propagates from one processing stage to the next. In turn, this leads to more pronounced slowing with each additional operation involved in processing of a task, which can account for slower and more erroneous performance in the switching trials compared to the repetition trials, as well as in the difficult task switching involving four operations compared to easy task switching involving two operations. However, besides the generally slower processing with age, no additional slowing in the switching trials with greater age shall emerge.

## Materials and methods

### Participants

The older participants of the study were drawn from the cohort of the study on Cardiovascular Disease, Living and Ageing in Halle (CARLA study, Greiser et al., 2005; Hassan et al., 2022). The CARLA Study is a population-based cohort study in the city of Halle (Saale), Germany. Its aim is to gather information on the prevalence and incidence of cardiovascular diseases, as well as their risk and prognostic factors, with a particular focus on age, based on a representative sample of the population in Halle (Saale) aged 45 to 83 years during the baseline examination. Of the original CARLA cohort, 342 participants gave their consent to be approached for participation in further studies. These participants were contacted via mail and received information about the purpose and procedure of the current study. In total, 179 participants replied and showed their potential interest in participation. After an additional telephone screening, we invited 133 participants ranging from 64 to 97 years in age (mean age of M = 75.28 years) to the lab at the Martin Luther University Halle-Wittenberg, where they took part in the current study. The final sample size of 133 participants was based on an *a priori* power analysis using G*Power software (Faul et al., 2007) to ensure sufficient power to detect an interaction between trial type and age group on switch costs. Assuming a small over-additive effect (η*p*^2^ = 0.03, *f* = 0.176), with α = 0.05 and power = 0.95, the required sample size was estimated at 129. The slightly larger final sample accounts for potential dropouts and is adequate to test whether age modulates switch costs. All participants were split into three age groups roughly based on the age classification of the World Health Organization (World Health Organization [WHO], 2015): the young-old group from 64 to 72 years (*N* = 51 participants, 31 male), the older-old group from 73 to 80 years (*N* = 51 participants, 34 male), and the oldest-old group from 82 to 97 years (*N* = 31 participants, 19 male). Written informed consent was collected before the start of the experimental session. All participants had normal or corrected-to-normal vision and received 15 euros as compensation for their participation. Approval of a local ethics committee (Ethic committee of Martin Luther University Halle-Wittenberg) was obtained before the commencement of the study. Two participants did not finish the experimental session and their data was excluded from further analyses. In Table 1, sample characteristics per age group of older adults are presented.

**TABLE 1:** Description of the population: means and standard deviations (in brackets) of age, gender, years of education, subjective ratings of multitasking ability (scale 1–5), body-mass index (BMI) for the young-old group, older-old group, and the oldest-old group.

	Age groups		
	Young-old group	Older-old group	Oldest-old group
Mean age (SD)	68.33 (0.34)	76.1 (0.31)	85.79 (0.70)
Gender	31 males, 20 females	34 males, 17 females	19 males, 12 females
Years of education (school and trainings)	14.93 (0.45)	16.21 (0.53)	14.82 (0.71)
Subjective rating of multitasking ability	3.25 (0.86)	3.14 (0.57)	2.86 (0.85)
BMI	28.35 (3.79)	28.27 (4.25)	27.71 (3.97)

Older participants were grouped into three age categories: young-old group (64–72 years), older-old group (73–80 years), and oldest-old group (82–97 years).

In addition, we recruited a group of younger participants (*N* = 36 participants, age range 19–33 years, M = 23.39, SD = 3.05) as a control group. Participants of this group were students of the Martin Luther University Halle-Wittenberg and they received a monetary compensation or a course credit for their participation in the experiment.

### Apparatus and tasks

The current study was part of larger research project that focuses on a variety of executive functions as well as their modulation due to age and other physiological measures. In particular, and in order to cover a wider range of executive functions (Miyake et al., 2000), participants also completed a numerical Stroop task as well as an auditory and a visual working memory updating task. In addition to the performance in the executive control tasks, various physiological measures, such as metabolic, inflammatory and cardiovascular screening data as well as sociodemographic data were obtained at earlier time points during the CARLA study (Greiser et al., 2005; Hassan et al., 2022). These data were included in other analyses and are reported elsewhere (Ebert et al., 2020; e.g., Efremov et al., 2020; Lacruz et al., 2018). Since in the current study we aimed to test for potential effects of older age on local switch costs, in the “Materials and methods” section we mainly focus on the description of the task switching paradigm.

Participants sat in front of a 24-inch LCD monitor with a resolution of 1920 × 1080 pixels and a refresh rate of 60 Hz. Timed stimulus presentation and data acquisition were controlled by using the PsychoPy software (Version 2022.2.5; Peirce et al., 2019). The stimuli of the tasks consisted of 2-digit numbers presented centrally on black a computer screen with a visual angle of 0.62° (*x*-axis) × 0.31° (*y*-axis) at a viewing distance of 80 cm. Responses were provided by pressing a corresponding number key on the numeric keypad of keyboard of the computer. Overall, participants performed four different tasks with the presented digit stimuli. The specific tasks were cued by the specific color of the target stimuli: an addition task (green), a subtraction task (red), a smaller digit plus 1 task (blue), and a larger digit minus 1 task (yellow). For the addition task, participants were asked to sum up the two presented digits (e.g., 52 = 7; please, note that, in order to avoid carry-over effects, the sum of the two digits never exceeded 9). The subtraction task required participants to subtract the smaller from the larger digit (e.g., 73 = 4). In the smaller digit plus 1 task, participants were instructed to add 1 to the smaller of the two digits (e.g., 37 = 6). For the larger digit minus 1 task, participants had to report the digit obtained by subtracting 1 from the larger of the two digits. For each task, participants were instructed to respond as quickly and accurately as possible.

### Design and procedure

**FIGURE 1:**
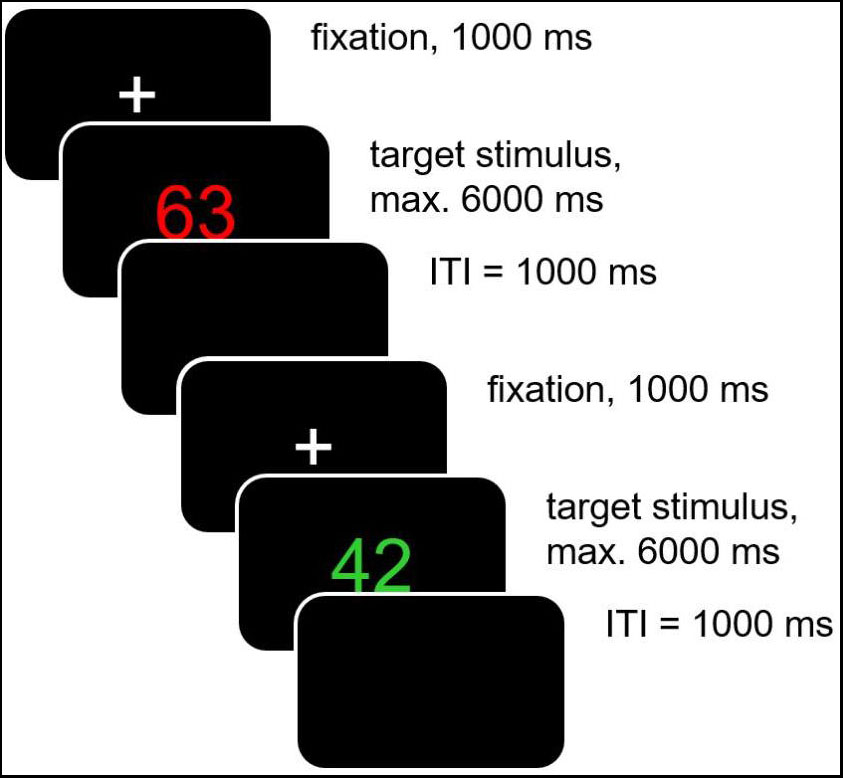
Details of the task switching paradigm applied in this study. In easy block I, target stimuli (two-digit numbers) were presented in either red or green in the upper half of the screen. For red target stimuli participants were instructed to sum up both digits, for green target stimuli to subtract the second from the first digit. In easy block II, target stimuli were presented in either yellow or blue in the lower half of the screen. For yellow target stimuli participants were instructed to add “1” to the smaller digit, for blue target stimuli to subtract “1” from the larger digit. In the difficult block, target stimuli were presented in red/green and yellow/blue in the upper and lower half of the screen, respectively, with the instructions used in easy block I and easy block II (please note that in all blocks only one target stimulus was presented per trial).

Each trial started with a fixation cross 0.31° (*x*-axis) × 0.31° (*y*-axis) which was presented for 1,000 ms (see Figure 1). Following the fixation cross, the 2-digit stimulus was presented until response or for a maximum response period of 6,000 ms. After an inter-trial-interval of 1,000 ms the next trial started.

Tasks were presented in two easy and in one difficult block. For the easy block I, participants either performed the *addition task* or the *subtraction task in different trials in varying order*. For the easy block II, participants switched between the *smaller digit plus 1 task* or the *larger digit minus 1 task*. Performance over the two easy blocks was averaged and included in further analyses as performance measure in the easy condition. To increase task difficulty, in the difficult block all four tasks were administered (Buchler et al., 2008). Each block consisted of 52 trials with 26 repetition and 26 switch trials. Participants first performed all three blocks in the following sequence: easy block I, easy block II, difficult block. Before each experimental block, participants received the instructions for the upcoming tasks as well as the relevant task rules and familiarized themselves with the stimulus material in practice block consisting of 27 trials.

Participants were invited to the lab of the research unit Experimental Psychology at Martin Luther University Halle-Wittenberg where they were tested individually in a single 90-min experimental session. Upon arriving to the lab, participants were introduced to the study, provided their informed consent and filled out a demographic questionnaire. After this, participants went through three different paradigms measuring three distinct sub-processes of cognitive control. A Stroop task paradigm, an auditory as well as a visual updating task and the described task switching paradigm for measuring inhibition, working memory updating and switching. Performing each paradigm took about 25 to 30 min, and, in order to avoid potential sequence effects, the order of these paradigms was counterbalanced according to a Latin square approach.

### Statistical analyses

For all analyses, practice trials and the first trial of each experimental block were excluded. Additionally, exclusively for analyses of RTs, trials with erroneous responses (discrimination errors) or omitted responses were discarded. Mean RTs and error rates were calculated for each combination of the factors trial type and difficulty condition. Overall, 7 participants produced missing values for at least one combination of the two factors. The method of *mean imputation* was used to estimate these missing data points. For this purpose, missing values were replaced by mean response times or error rates calculated for each combination of the factors trial type and difficulty. This resulted in imputation of 3.38% of cells. In addition to conventional response speed and accuracy measures, and, in order to control for potential speed-accuracy trade-offs, we also calculated and analyzed inversed efficiency scores as a compound measure of RTs and error rates (Townsend and Ashby, 1978; Vandierendonck, 2018). This is especially important, since research has shown that with greater age participants employ a more cautious processing strategy that prompts them to accumulate more information before making a decision which results in higher accuracy at the cost of decreased processing speed (Forstmann et al., 2011; Rabbitt, 1979; Salthouse, 1979).

To test whether or not age modulates switch costs, in the first step, we analyzed RTs, error rates, as well as inversed efficiency scores as a compound measure applying a mixed-measures ANOVA with the between-subjects factor age group (young group, young-old group, older-old group, oldest-old group) and the within-subjects factors difficulty (easy, difficult) and trial type (repetition, switch). Of particular interest for our research questions is the trial type × age group interactions, since this interaction is indicative of whether switch costs differ between the age groups or not. In order to provide further evidence for or against a modulatory effect of age on switch costs, we also applied a Bayesian mixed-measures ANOVA using JASP software (van den Bergh et al., 2020) comparing posterior probabilities of a model not including interaction terms indicating a difference in switch costs across all age groups with those of potential model allowing for a modulation of switch costs by age. A resulting Bayes factor BF_10_ with a value larger than 1 would provide evidence for the model including the interaction terms allowing for age-specific effects on switch costs, whereas a BF_10_ with a value smaller than 1 would provide evidence for a model not including these interaction terms. In a second step, we also conducted additional analyses employing linear regression modeling, in which we treated age as a continuous variable and not as grouping variable. The primary reason for this choice was to enhance statistical power and provide a more refined analysis. While the ANOVA assesses differences between predefined age groups, which requires a summarized conclusion about participants with different individual ages, linear regressions allow us to examine age as a continuous variable. This approach can help uncover more nuanced relationships that might be obscured when age is categorized by different groups. Thus, by modeling age continuously, we can more precisely quantify the relationship between age and switch costs and detect subtle effects that may not be apparent from the ANOVA results. In order to do so, we conducted a linear regression analysis to assess how age, modeled as a continuous variable, predicts RTs in repetition and switch trials, as well as switch costs.

## Results

### Effect of age group on performance in task switching

#### RTs

**FIGURE 2:**
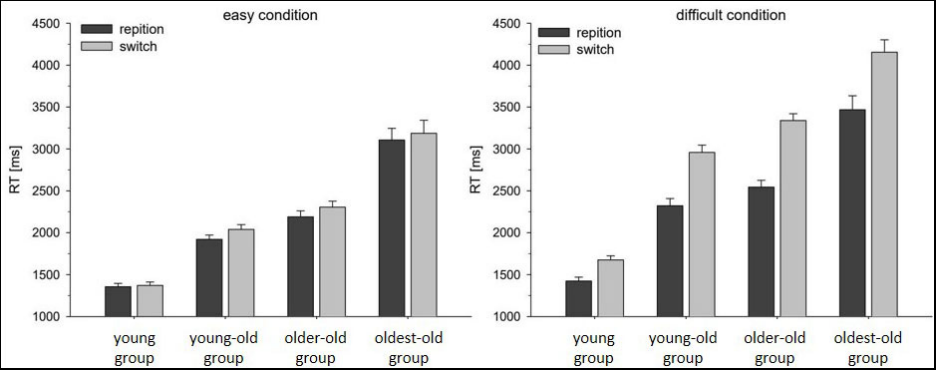
Mean RTs as a function of age group, difficulty condition and trial type. Error bars reflect the standard error of the mean. Left panel: RTs the easy condition, right panel: RTs for the difficult condition.

Analyzing mean RTs, we found an effect of the factor age group, *F*(3,163) = 92.15, *p* < 0.001, η_*p*_^2^ = 0.629. *Post-hoc* tests indicated that, in general, RTs differed between the young group (M = 1,457 ms) and the young-old group (M = 2,310 ms), *t*(85) = 9.92, *p* < 0.001, *d* = 2.16, between the young-old group (M = 2,310 ms) and the older-old group (M = 2,594 ms), *t*(99) = 3.05, *p* = 0.003, *d* = 0.61, and between the older-old group and the oldest-old group (M = 3,479 ms), *t*(78) = 6.40, *p* < 0.001, *d* = 1.48 (see Figure 2). In addition, participants produced longer responses in the difficult block (M = 2,731 ms) compared with easy blocks (M = 2,185 ms), *F*(1,163) = 305.79, *p* < 0.001, η_*p*_^2^ = 0.65. This effect of difficulty was more pronounced in the three older groups compared with the youngest group as illustrated by a significant interaction of age × difficulty on the RTs, *F*(3,163) = 14.18, *p* < 0.001, η_*p*_^2^ = 0.21. Furthermore, we found reliable switch costs, which were indicated by longer RTs in switch (M = 2,626 ms) compared with repetition (M = 2,290 ms) trials, *F*(1,163) = 386.81, *p* < 0.001, η_*p*_^2^ = 0.704. In addition, the switch costs (RT_*switch*_—RT_*repetition*_) were more pronounced under the difficult (M = 607 ms) versus the easy condition (M = 89 ms), as reflected by the difficulty × trial type interaction, *F*(1,163) = 227.11, *p* < 0.001, η_*p*_^2^ = 0.58. Crucially, and most important for the answer on the current research question, switch costs did differ between the age groups, as indicated by the significant interaction of the factors age and trial type, *F*(3,163) = 16.38, *p* < 0.001, η_*p*_^2^ = 0.23. *Post-hoc* tests revealed that switch costs for the young (M = 134 ms) group were smaller compared to the switch costs in the three groups of older adults: in the young-old group (M = 373 ms), *t*(85) = 6.58, *p* < 0.001, *d* = 1.43, in the older-old group (M = 455 ms), *t*(84) = 8.63, *p* < 0.001, *d* = 1.89, and in the oldest-old group (M = 383 ms), *t*(64) = 3.94, *p* < 0.001, *d* = 0.98. Moreover, among the groups of older adults, *post-hoc* tests revealed smaller switch costs in the group of young-old adults compared to the group of older-old participants, *t*(99) = 2.21, *p* = 0.029, *d* = 0.44, while no other differences between the groups were found significant (all *p* > 0.23).

Importantly, this pattern was more pronounced in the difficult condition compared to the easy condition, as reflected by the interaction of the factors age group, difficulty, and trial type, *F*(3,163) = 8.16, *p* < 0.001, η_*p*_^2^ = 0.13. This interaction was mainly driven by the fact that the difference in switch costs between the older groups (i.e., averaged across the three older groups) and the young group was larger in the difficult condition (M = 453 ms) compared with the easy condition (M = 92 ms), *t*(130) = 8.86, *p* < 0.001, *d* = 0.77. When it comes to differences in switch costs between the young-old group and the older-old group, they were not significant in the easy condition (the young-old group: M = 119 ms vs. the older-old group: M = 115 ms), *t*(99) = 0.45, *p* = 0.91, *d* = 0.024, but only in the difficult condition (the young-old group: M = 373 ms vs. the older-old group: M = 454 ms), *t*(99) = 2.59, *p* = 0.011, *d* = 0.91. These differences, however, were negligible after correction for multiple comparisons. For this reason, we conducted a Bayesian analysis, which again provided no evidence for larger switch costs in the young-old group vs. older-old group under the easy condition (*BF*_10_ = 1.79), but provided moderate evidence for larger switch costs in young-old group than in the older-old group in the difficult condition (*BF*_10_ = 3.96). No other differences between the age groups were found significant.

To sum up, we found an effect of age on switch costs, that is, with greater age the RTs increased more in switching trials than in repetition trials. The difference can be observed mostly when comparing switch costs between the group of young participants and the three groups of older participants, and to a lesser extent for the comparison between the group of young-old participants and the group of older-old participants. Finally, these age-related differences in switch costs were more pronounced in the difficult condition than in the easy task switching condition. Importantly, this pattern of results is in line with the hypothesis that aging leads to a specific impairment of executive control processes when treating age in a dichotomic manner by comparing the young group with the three groups of older participants (e.g., Phillips and Della Sala, 1998; West, 1996).

#### Error rates

Analyzing error rates, we found an effect of the factor age group, *F*(3,163) = 16.16, *p* < 0.001, η_*p*_^2^ = 0.23. *Post-hoc* tests indicated that, in general, the young group produced fewer errors (M = 4.9%) compared with the three older groups (all *p*s ≤ 0.001). Within the groups of older adults, error rates were lowerin the group of young-old adults (M = 16.4) than in the oldest-old group (M = 25.2%), *t*(79) = 2.74, *p* = 0.008, *d* = 0.63, while the error rates did not differ between the older-old group (M = 19.5%) and the oldest-old group, *t*(78) = 1.70, *p* = 0.093, *d* = 0.39 just as not between the young-old group and the older-old group, *t*(99) = 1.16, *p* = 0.25, *d* = 0.23. Participants produced more errors in the difficult block (M = 19.3%) compared with easy blocks (M = 13.7%), *F*(1,163) = 8.92, *p* = 0.003, η_*p*_^2^ = 0.052. This effect of difficulty did not differ between the three age groups, *F*(3,163) = 1.95, *p* = 0.123, η_*p*_^2^ = 0.035. Furthermore, error rates were larger in switch (M = 17.2%) versus repetition (M = 15.7%) trials, *F*(1,163) = 13.05, *p* < 0.001, η_*p*_^2^ = 0.074, indicating reliable switch costs in error rates. Additionally, the difference in error rates between switch and repetition trials was more pronounced in the difficult (M = 2.0%) relative to the easy condition (M = −0.1%), *F*(1,163) = 16.19, *p* < 0.001, η_*p*_^2^ = 0.09.

Crucially, and most importantly for the current research question, switch costs in error rate were not modulated by the factor age, as was indicated by the non-significant interaction of the factors age and trial type, *F*(3,163) = 1.05, *p* = 0.37, η_*p*_^2^ = 0.019. This lack of interaction was also supported by a Bayes factor of *BF*_10_ = 0.001 providing strong evidence for a model that does not include modulatory effects of age on participants’ switch costs in error rate. Importantly, this pattern remained also stable when distinguishing between easy and difficult blocks, as was reflected by the non-significant interaction of the factors age group, difficulty, and trial type, *F*(3,163) = 1.23, *p* = 0.30, η_*p*_^2^ = 0.022, *BF*_10_ < 0.001.

To sum up, only general performance, i.e., error rates in repetition and switch trials, but not local switch costs, were affected by age. Error rates can be found in Table 2.

**TABLE 2:** Mean rates of errors in % (and standard deviation) as a function of trial type, difficulty condition and age group.

	Error rates							
	Easy condition				Difficult condition			
	Age group				Age group			
Trial type	Young	Young-old	Older-old	Oldest-old	Young	Young-old	Older-old	Oldest-old
Repetition	4.6 (0.9)	10.3 (1.3)	16.7 (2.1)	23.4 (3.7)	4.2 (1.6)	20.2 (3.2)	21.5 (3.1)	25.4 (4.3)
Switch	4.9 (0.9)	10.3 (1.3)	16.4 (2.3)	22.9 (3.4)	5.8 (1.0)	24.9 (3.4)	23.2 (3.4)	29.3 (4.5)

Participants were grouped into following age categories: young group (18–30 years), young-old group (64–72 years), older-old group (73–80 years), and oldest-old group (82–97 years).

#### Inversed efficiency scores

To control for potential age-related biases in speed-accuracy trade-offs (Forstmann et al., 2011; Rabbitt, 1979) we analyzed inversed efficiency scores as a compound measure of RTs and error rates. They are illustrated in Table 3. We calculated the inversed efficiency scores according to Townsend and Ashby (1978; see also Vandierendonck, 2018) as RT divided by 1—proportion of errors for each type of trial and level of difficulty per age group. Analyzing inversed efficiency scores, we found an effect of the factor age group, *F*(3,163) = 22.59, *p* < 0.001, η_*p*_^2^ = 0.29. The youngest group produced smaller scores (M = 1,542) compared with all three older groups (all *p*s ≤ 0.001). Furthermore, the oldest-old group (M = 5,729) was the least efficient, as they produced larger scores compared with the young-old group (M = 3,322), *t*(79) = 3.78, *p* < 0.001, *d* = 0.87, and older-old group (M = 3,673), *t*(78) = 3.62, *p* < 0.001, *d* = 0.15, while the last group of young-old participants and of the older-old participants did not differ from each other, *t*(99) = 0.99, *p* = 0.32, *d* = 0.198. The differences remain also significant after controlling for multiple comparisons. In other words, the young group is still more efficient in task switching than all groups of older adults and the oldest-old group was shown to be the least efficient also when their RTs are adapted for the amount of errors they made. This suggests that the differences in performance cannot be attributed to some sort of speed-accuracy tradeoff of faster but more erroneous performance in young adults compared to the older adults. Also, inversed efficiency scores were higher in the difficult (M = 4,314) compared with the easy blocks (M = 2,813), *F*(1,163) = 28.69, *p* < 0.001, η_*p*_^2^ = 0.15. This effect of difficulty was tendentially larger in the young group than in the other groups, as the age × difficulty interaction was approaching significance, *F*(3,163) = 2.37, *p* = 0.07, η_*p*_^2^ = 0.042. In addition, switch trials (M = 3,199) yielded higher efficiency scores relative to repetition trials (M = 3,929), *F*(1,163) = 13.05, *p* < 0.001, η_*p*_^2^ = 0.074, resulting in reliable switch costs. Also, these switch costs, i.e., the difference between both trial types was larger in the easy (M = 32) compared with the difficult condition (M = 1,429), *F*(1,163) = 46.21, *p* < 0.001, η_*p*_^2^ = 0.22. Most importantly for the current research question, switch costs were modulated by the factor age, as was indicated by a significant interaction of the factors age and trial type, *F*(3,163) = 16.38, *p* < 0.001, η_*p*_^2^ = 0.23. *Post hoc* comparisons revealed that the group of young participants (M = 155) was significantly more efficient compared to the three groups of older participants: to the young-old group (M = 1,012 ms), *t*(85) = 3.05, *p* = 0.003, *d* = 0.66, to the older-old group (M = 846 ms), *t*(84) = 3.95, *p* < 0.001, *d* = 0.86, to the oldest-old group (M = 907 ms), *t*(64) = 3.28, *p* = 0.002, *d* = 0.81. No other differences between the groups of older adults were found significant (all *p*s > 0.55). In addition, we found a triple interaction of the factors age group, difficulty, and trial type, *F*(3,163) = 3.52, *p* = 0.016, M = η_*p*_^2^ = 0.13. This interaction was mainly driven by greater difference in the switch costs between the young group and all older groups in the difficulty (M = 1,758) compared with the easy condition (M = 42), *t*(130) = 6.74, *p* < 0.001, *d* = 0.59.

**TABLE 3:** Mean inversed efficiency scores (and standard deviation) as a function of trial type, difficulty condition and age group.

	Inversed efficiency scores							
	Easy condition				Difficult condition			
	Age group				Age group			
Trial type	Young	Young-old	Older-old	Oldest-old	Young	Young-old	Older-old	Oldest-old
Repetition	1,422 (40)	2,181 (81)	2,796 (168)	4,793 (666)	1,508 (60)	3,448 (366)	3,706 (289)	5,759 (844)
Switch	1,446 (42)	2,314 (86)	2,941 (169)	4,614 (506)	1,794 (64)	5,345 (772)	5,252 (463)	7,752 (1,236)

Participants were grouped into following age categories: young group (18–30 years), young-old group (64–72 years), older-old group (73–80 years), and oldest-old group (82–97 years).

Overall, and similarly to RTs, the analysis of the of the inversed efficiency scores provided again evidence for the assumption aging impacts to specific functions in cognitive processing (e.g., Phillips and Della Sala, 1998; West, 1996).

### Detailed analysis of age effects across the age continuum of older adults

**FIGURE 3:**
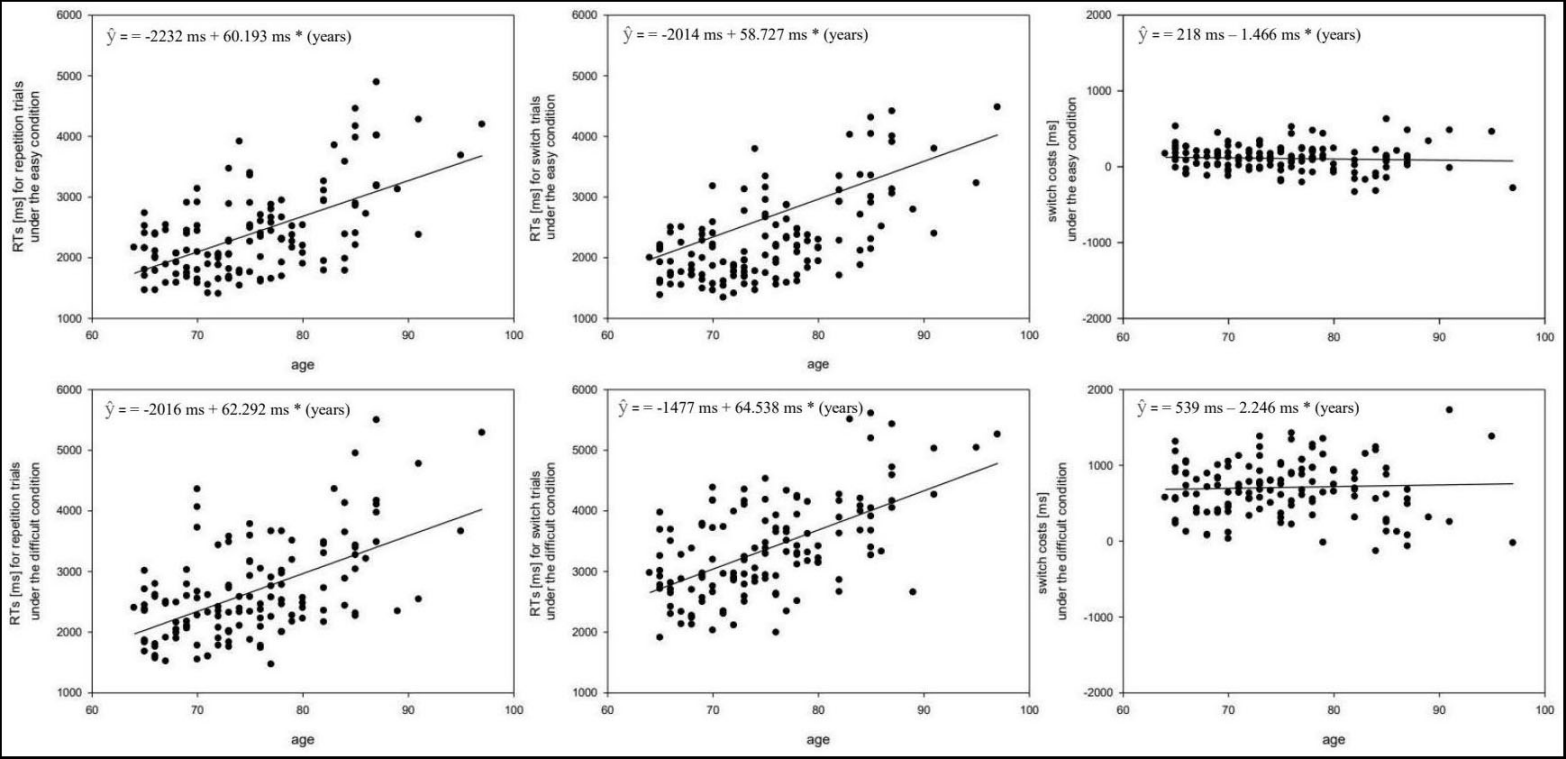
Regressions graphs with age as the predictor and RTs/switch costs as the criterium. Left column: RTs for repetition trials, middle column: RTs for switch trials, right column: switch costs. Upper row: easy condition, lower row: difficult condition.

We next examined the effect of age on older participants’ performance using linear regression, treating age as a continuous variable (64–97 years). This allowed for a more fine-grained analysis of age-related changes across the spectrum of older adults, beyond simple group comparisons. We tested whether age predicted RTs in repetition trials, switch trials, and switch costs. Figure 3 illustrates these results, showing RTs on the *y*-axis (repetition: left, switch: middle, switch costs: right) plotted against age (*x*-axis) for the easy (top row) and difficult (bottom row) conditions.

For the easy condition, age (for the range between 64 and 97 years) significantly predicted the mean RTs in repetition [*b* = 60.19, *t*(131) = 8.90, *p* < 0.001] and switch trials [*b* = 58.73, *t*(131) = 8.17, *p* < 0.001]. As can be seen in the graph, for both trial types RTs were longer with greater age in the range between 64 and 97 years. The overall regressions were significant for both repetition, *F*(1, 130) = 79.26, *p* < 0.001, *R*^2^ = 0.38, and switch trials, *F*(1, 130) = 66.78, *p* < 0.001, *R*^2^ = 0.34. However, the regression coefficients (b), for both regressions did not differ significantly, *z* = 0.416, *p* = 0.34, indicating a similar age-related slowing of RTs in repetition and switch trials. Importantly, while mean RTs were significantly affected by age, the switch costs, i.e., the difference in RTs between both trial types, were not affected by age. This was indicated by a slope of *b* = 1.47 close to zero, *t*(131) = 0.68, *p* < 0.496, as well as non-significant regression model, *F*(1, 130) = 0.47, *p* = 0.50, *R*^2^ = 0.004. Thus, when analyzing the age effect across the continuum between 64 and 97 years it turns out that greater age is related to longer processing times in repetition and switch trials to equal amount, reflecting an effect of age on general processing speed but not on switch costs.

The results for the difficult condition mirrored those of the easy condition. Age significantly predicted mean RTs for repetition [*b* = 62.29, *t*(131) = 7.54, *p* < 0.001] and switch trials [*b* = 64.54, *t*(131) = 8.19, *p* < 0.001]. As can be seen in the graph, for both trial types RTs were slower with greater age. The overall regressions were significant for both repetition, *F*(1, 130) = 79.26, *p* < 0.001, *R*^2^ = 0.306, and switch trials, *F*(1, 130) = 67.12, *p* < 0.001, *R*^2^ = 0.34. In addition, the slopes (b) for both regressions did not differ significantly, *z* = 0.382, *p* = 0.35, indicating similar age-related slowing in RTs for repetition and switch trials. Again, while mean RTs were significantly affected by age, switch costs, i.e., the difference in RTs between both trial types, were not affected by age. This was indicated by a slope of *b* = 2.246 that did not differ from zero, *t*(131) = 0.43, *p* = 0.67, as well as non-significant regression model, *F*(1, 130) = 0.18, *p* = 0.67, *R*^2^ = 0.001. Overall, across both difficulty conditions, when modeling age as a continuous variable and restricted in the age range between 64 and 97 years, we only find age effects on general response times but not on switch costs. Importantly, these results do not provide evidence for further specific impairment of the task switching function from young-old participants to the oldest-old participants but provide support for the general slowing hypothesis of cognitive aging when analyzing the age continuum from 64 to 97 years in more detail (Salthouse, 1981, 2004).

## General discussion

The current study aimed to address the inconsistent results on the effects of aging on local switch costs. For this purpose, we included three groups of older participants and extended the age range of the older adults compared to the age range in previous studies. In addition, to provide a more reliable test on the potential modulatory effect of age on switch costs, we asked participants to operate at the upper limit of cognitive capacity by including two difficulty levels. That is, we manipulated the number of task rules and applied an easy condition consisting of two task rules and a difficult condition comprising four task rules. Indeed, previous studies have shown that introducing such manipulations of difficulty can hamper switching efficiency and, thus, increase switch costs (see for example Monsell, 2003; Rubinstein et al., 2001; Wu et al., 2022). Thus, under conditions of a larger age range of older participants and increased cognitive demands, we expected greater age-related differences in local switch costs to emerge if these costs are susceptible to the effects of aging. In other words, if aging had a detrimental effect on flexibly switching between multiple tasks, potential differences in local switch costs between the different age groups should now come to light. In addition, these differences in switch costs might be more pronounced in the difficult compared to the easy condition. Such a pattern of results would be in line with the assumption that aging leads to specific decay in cognitive functions and in the current case, in executive control functions sub-serving task switching (e.g., Phillips and Della Sala, 1998; West, 1996). Alternatively, if this aspect of cognitive flexibility remains stable with age, as suggested by some of the previous studies (e.g., Chen and Hsieh, 2023), no differences in local switch costs between the age groups should emerge. At the same time, performance in switch and repetition trials would be expected to become slower to a similar extent with greater age of participants. Such a result pattern would be consistent with the processing speed hypothesis of age-related cognitive decline (Salthouse, 2019) which proposes that aging leads to generally impaired performance due to slower processing of information and, thus problems with encoding and retrieving the information in a timely manner.

As a main finding, we found larger local switch costs with greater age. However, this difference in terms of time was found mostly for the transition from the group of young adults to the first group of the older participants while there was no further change of switch costs across the age continuum. As expected, the obtained difference in switch cost between the group of young adults and the group of young-old adults was even more pronounced when the task difficulty was high, i.e., when the number of task rules increased from two to four. This finding was further supported by the inverse efficiency scores, as the difference in efficiency in local task switching between young and all groups of older participants also remained even when the response times are corrected for the number of errors. This suggests that the difference between young adults and older adults cannot be explained by a strategic shift in speed-accuracy tradeoff but represents indeed decay in cognitive flexibility with greater age.

The findings of the current study provide evidence for the assumption that local switch costs would become larger if the age range of older participants is wide enough (64–97 years old; Mayr, 2001). That is, the obtained differences between the young group and the older groups stand in line with the results of the study conducted by Kray et al. (2002), who showed that age effects on local switch costs depend on the difficulty of task switching, i.e., the number of different tasks. In that study, older participants (mean age 66.83 years old) and younger adults (mean age 23.06 years old) had to switch between two task rules within one block (easy condition) or between four task rules (difficult condition). The results revealed larger local switch costs in older adults compared to younger adults and these age-related differences were even more pronounced in the difficult condition compared to the easy one. It is noteworthy that in this study as well as in our study the pattern of task switching was not predictable, which might be a further factor contributing to the effect of the age differences in local switch costs (e.g., Kray et al., 2002). However, the current results extend the findings of Kray et al. (2002) in several aspects including age of participants and different manipulation of difficulty of task switching, which allows for a generalized conclusion about potential age effects on switch costs. That is, we compared the task switching performance of the young group with three groups of older adults instead of one group of older adults. Not only did we extend the age range of older participants, but the mean age in each of the groups of older adults in our study was above the mean age of the older group included in the study by Kray et al. (2002), that is, above average age of 65. Besides this, in the previous study participants were presented with words as stimuli and were required to switch between four different categorization tasks (i.e., animacy, number of syllables, number of letters, presence of the letter “H”), while in the present experiment participants were presented with digits and were asked to switch between four arithmetic tasks (i.e., adding or subtracting from a larger or a smaller number). Despite the number of component tasks in the difficult condition being the same, arithmetic component tasks included in the present study are more cognitively demanding compared to the categorization tasks and reduce potential differences in reading ability among participants that could have contributed to the result pattern in the study of Kray et al. (2002). Taken together the increased number of component tasks in addition to the cognitive demands of the arithmetic operations of these component tasks, we argue that the difficulty manipulation was even higher compared to the previous studies and that older groups of participants in our study were indeed pushed to operate at their upper cognitive limit. Thus, also for this different set of component tasks we found differences in the local switch costs between the group of young adults and three groups of older adults. Moreover, we showed even with an increased task difficulty local switch costs remain relatively stable over the age range between 64 and 97 years. This latter aspect will be discussed in more detail below.

Besides the differences in local switch costs between the young group and the older groups (collapsed), we also investigated potential differences in switch costs across the whole age continuum of the older adults. When focusing on the groups of older adults, the pattern of changes in local switch costs becomes less clear. On the one hand, we found preliminary evidence that local switch costs might be larger at greater ages, i.e., beyond the age of 64 years. Namely, we found smaller switch costs in terms of time in the group of young-old adults than in the group of older-old adults. This difference seems driven by high task difficulty, as no difference in switch cost between these two groups was obtained in the easy condition. However, we note that this finding shall be taken with caution, as the Bayesian factor revealed only moderate support for the difference between the young-old participants and older-old participants in the difficult condition. Moreover, the pattern of results obtained in switch costs in terms of error rates did not follow the one observed in time costs, as no differences were found among the groups of older adults. Thus, to investigate these differences between the groups of older adults in more detail, we conducted a series of regression analyses with age as a continuous variable and response times in the switching and repetition trials. The results of these analyses did not provide evidence for a additional differences in local switch costs, meaning that the age-related changes in response times was the same for both repetition and switching trials.

Interestingly, the analysis of inverse efficiency scores revealed that the oldest-old group of participants is less efficient in terms of switch costs compared to the other groups. This finding suggests that local switch costs might be larger at an even older age (i.e., above age of 82), but the differences start to come to light only when the strategic shifts in terms of the speed-accuracy tradeoff are controlled. However, this effect shall be taken with caution, as this oldest-old group was the smallest and the variability in this group was larger compared to the other groups. Thus, how local switch costs are modulated at even older age remains to be further investigated in future studies. Nevertheless, the current approach to extend the age range for investigating potential age effects is promising in order to disclose potential age effects on executive functions.

Taken together, our findings stand in line with the assumption that under conditions of a sufficiently large age range of participants and task difficulty age-related changes in local switch costs will start to emerge. Despite that age-related changes are not taking place at same rate between the groups of young and young-old adults as between the groups of young-old adults and the oldest-old adults, these results are generally in accord with the group of theories assuming that aging affects specific executive processes to a larger extent than others (e.g., Hasher and Zacks, 1988; Mayr, 2001). In the context of the current study, this means that aging affects particularly the processes of flexible activation and de-activation of task representations required in the switching trials compared to the repetition trials. This age-related decay of specific cognitive functions can be explained by the differential effects of aging on separate subsystems within the frontal cortex, which is in line with the frontal aging hypothesis (e.g., Dempster, 1992; Greenwood, 2000; Phillips and Della Sala, 1998; West, 1996, 2000). According to this view, prefrontal regions are especially susceptible to the effects of aging, while other brain regions (e.g., visual cortex) show little or smaller changes (e.g., Dempster, 1992; West, 1996). Selective changes in prefrontal regions in terms of structure and function can, thus, account for the age-related cognitive loss observed in specific aspects of cognitive performance as these different cognitive functions rely on separate neural correlates in the prefrontal cortex (Braver et al., 2003; Goffaux, et al., 2006) and are differently sensitive to the effects of aging (Kray and Lindenberger, 2000; Mayr, 2001). Our results provide evidence for process-specific decline in local switch costs due to aging, the effect that cannot be explained by a general slowdown in perception and processing.

However, this specific difference in greater local switch costs mainly resulted from the contrast between the group of young adults in comparison to the collapsed data of the three groups of the older adults. For the first time to the best of our knowledge, it was shown that these specific changes seem to reach a plateau and remain constant above the age of 65. Above this age, we provided weak evidence for further specific decay in local switch costs, but clearly observed only further decay in response times, which was of the similar extent in both switching and repetition trials. This indicates that, from a more differentiated view of the age-related development, the changes in switch costs among the different age groups of older adults correspond to the general slowing down which stands in line with the processing speed hypothesis (e.g., Salthouse, 1993, 1996). Thus, our results point out that the theoretical accounts of specific decay and general slowing are not mutually exclusive, however their relative contributions seem to differ at different at different stages of cognitive aging in task-switching. That is, specific decay in local switch costs seems to play a pivotal role between young age and young-old age, while general slowing predominantly drives the age differences above age of 65. Taken together, the present study contributes to the growing body of research suggesting that both specific decay as well as general slowing down seem to play a role in age related cognitive decline to a different amount and with different time scales across the lifespan (Albinet et al., 2012).

Future studies shall further investigate the role of both specific decay and general slowing in the switch costs. The current results strongly suggest that increasing difficulty as well as age-range of older participants, as well as including various measures and analyses to assess these effects (i.e., age as categorical and continuous variable, inversed efficiency scores) is a fruitful approach to gain more fine-grained picture of age-related changes. This highlights the importance of greater integration of different approaches in future research. Also, in the present study we wanted to specifically focus on cognitive flexibility aspect of task switching and did not address global switch costs. Thus, such an approach for investigating age-related changes in global switch costs remains to be still to be implemented. Moreover, our results suggest that specific decay in local task-switching function takes place between young group and the young-old group. Thus, in future studies including also middle-aged participants could provide a clear picture about the trajectories of change in the local task switching performance, as well as contributions of specific deficits between young group, middle-aged group, and old groups. In addition, neuropsychological changes in the recruitment of PFC during easy and difficult task switching in young adults and wider age-range of older participants could provide further insight into differences in processing efficiency of PFC. Such studies could provide a valuable evidence for fine-grained age-related changes of ERP components (e.g., P300) over wider age-range between low and high difficulty. Namely, previous studies already indicated that differences in ERP components between two levels of task difficulty are more pronounced in younger adults than in the older adults (e.g., Guerrero et al., 2022) indicating generally less efficient and compensatory processing of the older adults. Finally, the current study implemented a cross-sectional design to investigate age-related changes in local task switching. Thus, future studies shall provide further insights by including longitudinal design and address to which extent individual age-related changes in both behavioral performance and electrophysiological measures can be attributed to specific deficits and general slowdown hypotheses.

Cognitive flexibility plays a pivotal role in adaptive functioning, and its decline with age has broad clinical implications — from reduced independence in daily life, to early detection of neurodegenerative conditions and diminished treatment responsiveness. Our results suggest that response times in difficult task conditions are particularly sensitive indicator of specific age-related changes in cognitive flexibility and differentiation between the age groups. Critically, such slowing, especially under higher cognitive work-load of switching between several goals in memory, puts older adults at higher risk especially in situations where quick responses are needed (e.g., driving a car). As such, understanding and fine-grained tracking changes in flexibility may aid in identifying older adults at higher risk for functional decline and help guide the development of targeted interventions aimed at maintaining cognitive adaptability and quality of life.

## Data Availability

The data that support the findings of this study are available at the following link: https://osf.io/m5z4u/?view_only=42ac8f85a7914df4b3aa5368ae69924f.
